# Supplementary material for: The interactome of intact mitochondria by cross-linking mass spectrometry provides evidence for coexisting respiratory supercomplexes
Source: Mol Cell Proteomics. 2017 Dec 8;17(2):216–32. doi: 10.1074/mcp.RA117.000470 (PMC5795388; doi:10.1074/mcp.RA117.000470)
Supplement: Supplemental Data [file supp_RA117.000470_133922_0_supp_23978_fzffwf.zip › spectra_annotation/mito_spectra_annotation/385-1-17-1-14-1.pdf]

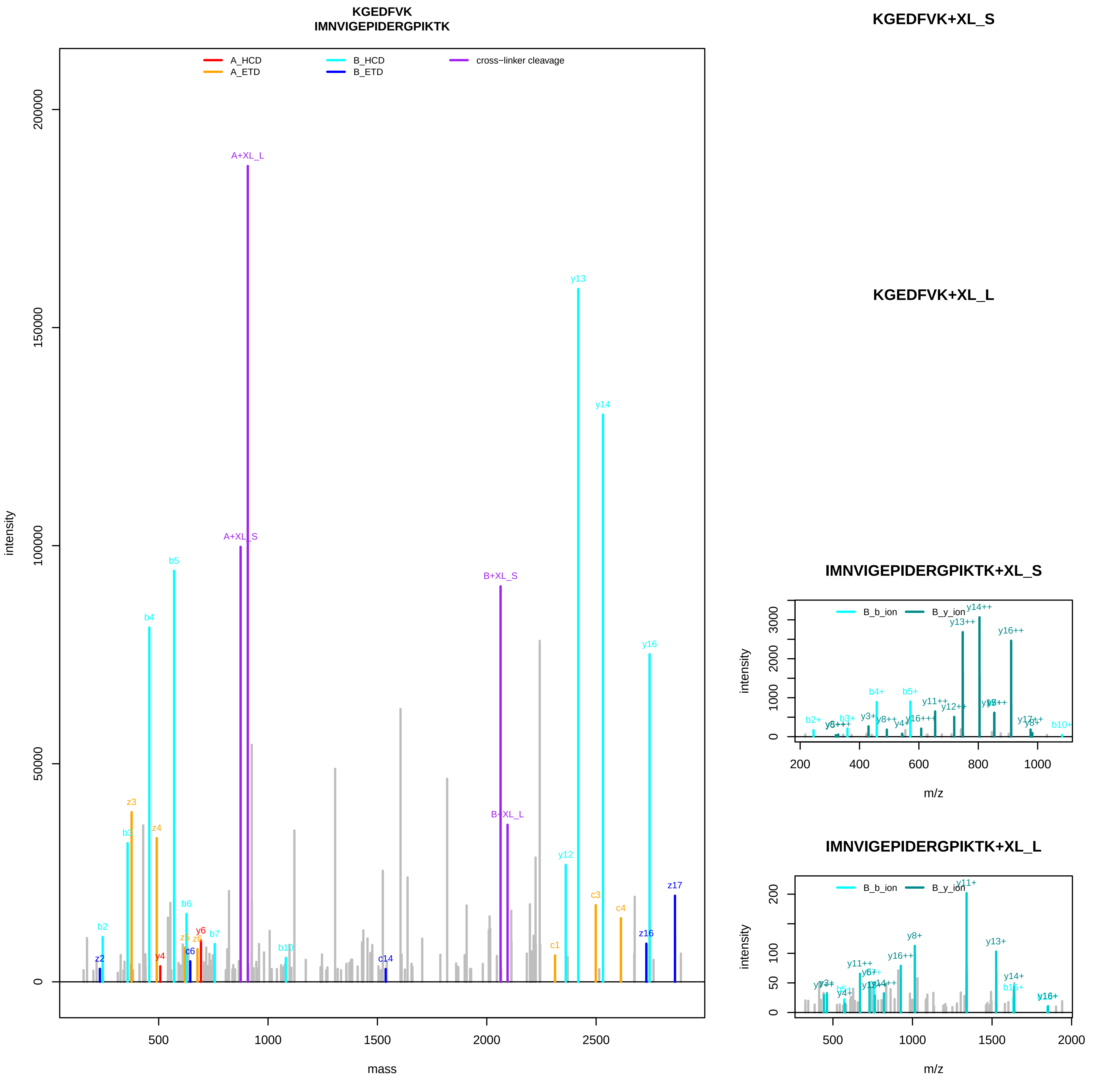
KGEDFVK
IMNVIGEPIDERGPIKTK
A_HCD
A_ETD
B_HCD
B_ETD
cross−linker cleavage
intensity
mass
A+XL_L
A+XL_S
B+XL_S
B+XL_L
y13
y14
y16
y12
z17
z16
c3
c4
c1
b5
b4
b3
z3
z4
b6
b7
y6
y4
z2
b2
c6
z5
z6
b10
c14
KGEDFVK+XL_S
KGEDFVK+XL_L
IMNVIGEPIDERGPIKTK+XL_S
B_b_ion
B_y_ion
y14++
y13++
y16++
b4+
b5+
y11++
y12++
b2+
b3+
y3+
y4+
y17++
b10+
m/z
IMNVIGEPIDERGPIKTK+XL_L
y11+
y8+
y13+
y16++
y11++
y14+
b15+
y16+
y4+
b5+
y3+
